# Longitudinal relations of prematurity and fetal growth restrictions with hyperactivity/inattention and aggression/delinquency

**DOI:** 10.1038/s41390-024-03297-y

**Published:** 2024-06-19

**Authors:** Jelena Zumbach-Basu, Annika Rademacher, Ute Koglin, Doris Bender, Friedrich Lösel

**Affiliations:** 1 Department of Forensic Psychology, Psychologische Hochschule Berlin, Germany, Am Koellnischen Park 2, 10179 Berlin, Germany; 2 I. School of Education and Social Science, Department of Special Needs Education and Rehabilitation, Psychology in Special Needs Education and Rehabilitation, Carl von Ossietzky Universität Oldenburg, Ammerlaender Heerstrasse 114–118, 26129 Oldenburg, Germany; 3 Department of Psychology, Friedrich-Alexander-University Erlangen-Nuremberg, Bismarckstr. 6, 91054 Erlangen, Germany; 4 Institute of Criminology, Cambridge University & Department of Psychology, Friedrich-Alexander-University Erlangen-Nuremberg, Sidgwick Avenue Cambridge CB3 9DA & Bismarckstr, 1, 91054 Erlangen, Germany

## Abstract

**Background:**

It is the aim of this study to analyze the longitudinal relationship between premature birth and low birth weight and the reciprocal influence between hyperactive/inattentive behavior and aggressive/delinquent behavior problems in children from early to late childhood.

**Methods:**

This study contains data from the German Erlangen-Nuremberg Development and Prevention Study. It applies prospective longitudinal path analyses on data obtained from postnatal pediatric assessments as well as later psychosocial behavior assessments by teachers and parents on *N* = 667 children, out of which *n* = 83 children (12.44%) were born preterm/small for gestational age.

**Results:**

The results show direct effects of birth complications at the beginning of preschool on hyperactivity/inattentiveness (teacher rating: *ß* = 0.28; *p* = 0.017; parent rating: *ß* = 0.32; *p* = 0.005), but not on aggression/delinquency (teacher rating: *ß* = 0.002; *p* = 0.427; parent rating: *ß* = 0.12; *p* = 0.324). Reciprocal effects between aggression/delinquency and hyperactivity/inattentiveness were stable at the end of elementary school, but not at the end of preschool across informants.

**Conclusion:**

Our results support a differentiated view on the potential development of behavior problems after birth complications and the demand for early prevention measures.

**Impact Statement:**

- Our results extend to the existing body of research by providing insight into the longitudinal effects of prematurity and fetal growth restrictions on hyperactive and aggressive/delinquent behavioral problems throughout a rather long period of development in childhood.
- The results show direct effects of birth complications on the development of hyperactivity for boys, but not for girls across informants. No direct effects of birth complications on aggression/delinquency are found.
- Our findings speak against too simple views on behavioral consequences of birth complications and thus can relieve too anxious parents, however close monitoring of the behavioral development of respective children is indicated.

## Objective and background

The important role of psychosocial factors in the development of antisocial and aggressive behavior has long been recognized. In recent decades, interest in the neurobiological bases of aggression has increased.^1–5^ However, in explaining persistent antisocial behavior, we often find a polarization between social and biological views in criminology. Developmental theories that address the persistence of antisocial behavior across the lifespan focus on early developmental factors and assume that the interaction of traits and environmental factors leads to antisocial continuity (Moffitt, 1993; Moffitt, 2005).^6,7^

A body of research aims to examine early neurodevelopmental risk factors for externalizing and antisocial behavior in later development.^8–12^ To date, however, many questions remain unresolved on how biological or neuropsychological risk factors affect later development when combined with psychosocial risks, leading to calls for a more integrated biopsychosocial perspective.^13,14^

Neuropsychological risks can be associated with birth complications, such as premature birth and low birth weight. These early life neuropsychological risk factors appear to have an impact on the neuroregulatory systems, mediating aggression. It is assumed that pregnancy and birth complications can influence neurological development in the child, leading to deficits in executive functions, impulsivity (less self-control), or lower intelligence. These deficits in turn are associated with behavior problems linked to aggressive and antisocial behavior.^8,11,15–18^

A substantial body of research supports links between childhood hyperactivity and juvenile aggression.^19–22^ Impulsivity, inattentiveness to instructions, and inability to retain information and limitations in the ability to think rationally through the likely consequences of actions have been found as criminogenic factors.^23–27^ Self-control may also be a protective factor that buffers other risks in the development of aggressive behavior.^28^ However, how birth complications lead to later adverse behavioral outcomes via neuropsychological deficits appears to be a complex and multidimensional process.^10^ Empirical findings on whether birth complications, such as premature birth and low birth weight predict aggressive and violent behavior directly or only in combination with other neuropsychological or psychosocial risk factors are mixed.

A number of studies report direct relations between birth complications and behavior problems, partly mediated by IQ or moderated by sex of the child. Klein et al.^29^ report that children born preterm showed higher attention problems scores at toddler age than children born full-term without medical problems.^29^ Domeloff et al. (2020) found that pre-term born children showed significantly lower cognitive performance and higher attention-deficit/hyperactivity problem scores at age 7 to 8 years, when compared to children born full-term. Global cognitive functioning did not predict behavioral outcomes independently, but a lower parental educational level independently contributed to increased risk for poorer cognitive and behavioral functioning in children born pre-term.^30^ Harvey et al.^31^ report that children showing hyperactive behavior and oppositional defiant behavior had more prenatal/perinatal birth complications and a greater family history of hyperactivity than did non-problem children. The authors conclude that as early as age 3, these behavior subtypes appear to be linked to biologically-based risk-factors.^31^

In the study conducted by Liu et al.^11^, birth complications had a direct effect on child externalizing behavior at age 11 years with a mediating effect of IQ on this relationship. Neither birth complications nor malnutrition interacted with psychosocial adversity in predicting childhood externalizing behavior. According to the authors, this indicates that birth complications result in brain dysfunction, as reflected by low IQ, which in turn predisposes to behavior problems.^11^

In a study conducted by Jackson and Vaughn.^32^, a greater number of maternal medical risks during pregnancy increased the odds of childhood externalizing behavior, but only among male offspring.^32^ Analyzing a sample of monozygotic twins, Mollegaard.^33^ report that birth weight was significantly negatively associated with emotional problems among girls and significantly negatively associated with hyperactivity among boys at age 12 years.^33^ These findings are in accordance with the general literature showing that under difficult bio-psycho-social conditions in childhood and youth, boys exhibit more externalizing and girls more internalizing behavioral problems.^34,35^ The study conducted by Taylor et al.^36^ stands out by analyzing the stability of behavior problems in extremely low birth weight ( < 1000 g) adolescents through more than one point of measurement (at age 8 years and at age 14 years). Children born with extremely low birth weight had higher symptom severity scores in parent ratings at age 14 years than normal birth weight controls for inattentive attention-deficit hyperactivity disorder (ADHD), anxiety, and social behavior problems. Group differences in parent ratings decreased over time for ADHD, especially among females, but were stable for anxiety and social behavior problems.^36^

Some studies point to an interaction of pregnancy and birth complications with family risks, such as a lower socio-economic status or inadequate parenting behavior.^8,15,37,38^ A few studies report direct effects of psychosocial factors, such as smoking during pregnancy, teenage motherhood, maternal depression, and maternal socio-economic status on behavioral problems and criminal offending, but no direct effects of perinatal factors, such as low birth weight or preterm gestational age.^39–41^ Accordingly, Koglin and Lösel.^13^ only found a significant effect of birth complications on externalizing child problems in low SES families.^13^

Overall, the above-mentioned and other research shows that pregnancy and birth complications can have an influence on the development of behavioral problems in childhood and adolescence. However, the findings are heterogeneous and, as in other fields of criminology and developmental psychopathology, need more replications.^42^ For example, there is variation with regard to direct effects or interactions with the sex of the children, type of outcome, measurement of variables, social factors, and study design. In particular, questions remain regarding the specific mechanisms of how birth complications lead to the emergence of aggressive and delinquent behavioral outcomes and the role of hyperactivity and attention problems or sex of the child within this process. Especially, there is a need for prospective longitudinal studies that include perinatal risk factors as well as hyperactivity and aggression and examine the stability of behavioral problems across several points of measurement.

It is the aim of this study to analyze the longitudinal relationship between birth complications and potential reciprocal influences between hyperactive/inattentive behavior and aggressive/delinquent behavior problems in children across three time points: in early childhood when children start preschool, in middle childhood when children finish preschool, and in late childhood when children finish elementary school. We analyze cross-lagged effects between hyperactivity/attention problems and aggressiveness/delinquency, taking into account the stability of both types of behavior problems (autoregressive effects). Our results extend to the existing body of research by providing insight into the longitudinal effects of birth complications on behavioral problems throughout a rather long period of development in childhood.

## Method

### Sample and procedure

This article contains data from the German Erlangen-Nuremberg Development and Prevention Study, using pediatric data on birth complications and various systematic assessments of externalizing behavioral problems. Data was collected in a longitudinal design from childhood through early adolescence.^43–45^ The sample consisted of 667 children (334 boys, 333 girls) from 602 families (8 cases with missing postnatal pediatric exam record data were excluded). Children were between three and five years old when entering the study (age *M* = 56.44 months; *SD* = 9.3 months). The participants were recruited from children’s kindergartens and preschools that were randomly selected in the area of Erlangen-Nuremberg, Germany. Apart from sufficient German language skills of parents to complete the assessment instruments, we applied no further inclusion criteria. Depending on the varying cooperation by parents and institutions, as normally, some self-selection may have occurred, but the socio-economic structure in the area was properly represented in the final sample.^45^

In this study, we use data that was obtained at the first point of measurement when children started preschool (T1), at the second point of measurement (T2) when children finished preschool (age *M* = 67.7 months; *SD* = 9.7 months), and at a third point of measurement (T3) when children finished elementary school (age *M* = 126.9 months; *SD* = 10.65 months). Information on birth complications was collected based on children’s postnatal pediatric assessment record in the repeated regular early diagnosis system of Germany. The included data are based on direct medical assessments. The German pediatric documentation starts after birth and contains 9–11 assessments in childhood. Unfortunately, not all parents are regularly showing up in pediatric practices so that participation rates decline over time. However, parents are reminded if they miss a date and the earliest assessments on which our data on birth complications based, are taken by nearly all parents. Participation in our study was voluntary and informed consent and written parental permission were obtained. The study was approved by the German Federal Ministry of Family Affairs and the Research Ethics Committee of the University of Erlangen-Nuremberg.

The socio-economic status of the families was measured by an index integrating income, educational level, profession, and housing conditions of the family.^46^ According to this index, 12.89% of the families of the total sample were lower class, 32.15% lower middle class, 35.56% middle class, 15.7% upper middle class, and 3.7% upper class. This distribution is in accordance with the rather good economy and level of education in Franconia (Bavaria, Germany), where we collected our data.

### Measures

#### Birth complications

Information on gestational age and birth weight was collected in home visits by a member of the research team from the postnatal pediatric assessment record data booklet. In Germany, this data is routinely documented by pediatricians in preventive examinations after birth and throughout childhood.

For our analyses, birth complications were defined as prematurity and/or growth restriction. For determining growth restriction, we followed the common definition as a weight below the 10^th^ percentile for the gestational age (small for gestational age [SGA]).^47^ Premature children were defined as children born before 37 weeks of pregnancy were completed.^48^ We identified 83 children (12.44%) in our sample that experienced such birth complications. The sample distribution of birth complications is displayed in Table 1. The very few children with severe malformations, disabilities, or chromosomal abnormalities were not included in the study, to avoid confounding.

**Table 1:** Sample distribution of birth complications.

	*N* (%)
Children born maturely/AGA	584 (87.56%)
Children born prematurely ( < 37 weeks of pregnancy)	33 (4.95%)
Children born SGA ≤10th percentile	35 (5.25%)
Children born SGA ≤3rd percentile	10 (1.50%)
Children born both, prematurely and SGA	5 (0.75%)
Total	667 (100%)

*AGA* appropriate for gestational age, *SGA* small for gestational age.

Further health-related information at birth was obtained on type of delivery, Apgar-scores at 5 and at 10 min after birth, cord arterial pH-scores, and length of hospital stay after birth. We report this data for a more detailed description of the health status of our sample at birth.

#### Behavioral problems of the children

A German version of the “Social Behavior Questionnaire“ (SBQ).^49^ was used to measure behavioral problems of the children in preschool teacher and parent ratings (T1 and T2) and in school teacher and parent ratings (T3). The teachers and the parents individually filled out the symptom lists and were instructed to rate how the child behaved or felt over the last 12 months. According to the traditional German educational policy of unpaid education, preschool and elementary teachers came from public schools, although there is a recent trend toward more private schools.

The SBQ is widely used internationally in research and practice. It contains a standardized instruction. As in most research studies using the SBQ, no further training on the administration of the behavior rating scales seemed to be necessary. It would also have been impractical because the informants came from many different institutions and we did not address single cases in daily practice. Like in other larger research studies, we emphasize that scale differences in behavior problems are continuous and do not indicate clinical dichotomies between individuals. This is in agreement with developments in ICD, DSM, and also in child and youth psychiatry, where problems are more often seen as continuum instead of a taxonomic dichotomy.^50^ The SBQ assessment consists of 47 items on children’s behavior that are rated on a three-point scale: “not applicable/never” (score 0), “sometimes applicable/sometimes” (score 1), and “mostly applicable/almost always” (score 2). We examined the behavioral outcome in two dependent variables. The first dependent variable was a combined scale on “physical aggression” and “destruction/delinquency”. We combined both scales to get more inter-individual variation at younger age when specific symptoms were rare. The scale “aggression/delinquency” consists of 11 items; (Cronbach’s alpha = 0.88 in our study). Item samples are “hit, bit, or kicked other children”; “destroyed things that belong to others”; “stealed”. As second dependent variable, we used the SBQ-scale “hyperactivity/inattentiveness”. This scale consists of 8 items (Cronbach’s alpha = 0.90). Typical items are “was impulsive, acted without thinking” and “had difficulties to focus on one thing at a time”.

### Statistical analysis

We descriptively analyze information on the health-related variables of our sample at birth. Regarding behavioral problems, we report means, standard deviations and intraclass correlation coefficients (ICCs) for teacher and parent ratings on hyperactivity/inattentiveness and aggression/delinquency at T1, T2, and T3.

For the subsequent path analysis, we refrain from combining parent and teacher ratings. Literature on comparing methods of integrating parent and teacher symptom ratings provide mixed results, indicating that no single method of combining ratings is superior in identifying impaired children.^51,52^

All subsequent path analyses are based on teacher ratings, and parent rating respectively. Regarding hyperactivity and ADHD, teacher reports tend to capture more severe expressions,^53^ as these are more likely to become evident in a school setting when attention and activity related requirements are higher. Parent ratings on the other hand tend to be superior in identifying globally impaired children.^52^ Prior to path analysis, all scales were z-transformed.

Direct and indirect associations of birth complications (prematurity/growth restrictions), hyperactive/inattentive behavior and aggressive behavior/delinquency at the beginning of preschool, at the end of preschool, and at the end of elementary school were examined in different path models, based on teacher ratings and on parent ratings respectively. Cross-lagged path models allow for the estimation of longitudinal effects, controlling for correlations within time points, and controlling for the stability of variables over time by considering autoregressive effects. These models can provide preliminary evidence for causality in non-experimental data.^54^

Birth complications (prematurity/growth restrictions) were examined as a predictor for hyperactive/inattentive and aggressive/delinquent behavior problems. Cases with single missing data were estimated within the models. The model fit indices for the path analyses were the *chi*^*2*^ value, the *root mean squared error of approximation* (RMSEA), the *comparative fit index* (CFI) and the *Tucker-Lewis index* (TLI). *CFI* and *TLI* values > 0.90 represent good model fit.^55^ According to Hu and Bentler,^55^ RMSEA should reach values < 0.08 to represent good model fit. However, Kenny et al.^56^ indicate that using the RMSEA to assess the model fit in models with small degrees of freedom is problematic and potentially misleading. We therefore report RMSEA values for our models, but avoid interpretation using cutoffs. To account for non-normal distributions of our outcome variables, we ensured that replicating our analyses using a bootstrapping technique with 50 replications produced stable results.^57^ The analyses were conducted with STATA (Version 15).

## Results

Descriptive statistics on children’s gestational age and weight, as well as on further health related variables (Apgar-scores at 5 and at 10 min after birth, cord arterial pH-scores) are displayed in Table 2. Children were born via vaginal delivery (77.06%), Cesarian section (15.29%), and vaginal surgery (6.45%). Length of hospital stay after birth varied from less than one week (71.96%), over one week (2.55%), two weeks (0.75%), up to four or more weeks (0.15%).

**Table 2:** Descriptive statistics on birth complications (predictor variables) and further health-related variables.

	Children born prematurely/SGA (*n* = 83)					Children born maturely/AGA (*n* = 584)				
	M	SD	Md (25%, 75% perc.)	Min	Max	M	SD	Md (25%, 75% perc.)	Min	Max
Gestational Age & Weight (Predictor Variables)										
Gestational age	37.46	2.59	38 (36, 40)	30	41	39.74	1.14	40 (39, 40)	37	42
Gestational weight in grams	2497.76	447.75	2550 (2300, 2760)	1370	3950	3468.69	379.22	3450 (3200, 3700)	2560	4800
Further Health-Related Variables at Birth										
Apgar-score at 5 min	9.40	0.98	10 (9,10)	5	10	9.80	0.63	10 (10, 10)	1	10
Apgar-score at 10 min	9.75	0.73	10 (10, 10)	5	10	9.93	0.36	10 (10, 10)	4	10
Cord arterial pH-score	7.24	0.11	7.26 (7.2, 7.3)	6.9	7.66	7.28	0.12	7.28 (7.23, 7.33)	6.98	7.55

*AGA* appropriate for gestational age, *SGA* small for gestational age.

Table 3 presents descriptive statistics and intraclass correlations between teacher and parent ratings on behavioral variables. Intraclass correlations ranging between *ICC* = 0.10 and *ICC* = .39 indicate a low to moderate agreement across informants. The descriptive statistics and inter-correlations for all variables included in the Path Model 1 (teacher rating) and Path Model 2 (parent rating) are displayed in Tables 4, 5. Significant correlations between the independent and dependent variables show a relationship between prematurity/growth restrictions and hyperactive/inattentive behavior problems at T1 in both, teacher and parent ratings. Further significant correlations consistently exist between hyperactive/inattentive and aggressive/delinquent behavior problems across informants. All of the examined behavioral problems were significantly associated with the sex of the child.

**Table 3:** Descriptive statistics and intraclass correlations between teacher and parent ratings on behavioral variables.

	Teacher M (SD)	Parent M (SD)	ICC (95% CI)
Hyperactivity/ Inattentiveness T1	3.76 (3.96)	4.93 (3.47)	0.33 (0.26-0.39)***
Hyperactivity/ Inattentiveness T2	3.39 (3.94)	4.64 (3.67)	0.39 (0.32–0.45)***
Hyperactivity/ Inattentiveness T3	3.16 (3.87)	4.42 (3.44)	0.29 (0.19-0.36)***
Aggression/ Delinquency T1	1.83 (2.27)	2.27 (1.87)	0.31 (0.23-0.37)***
Aggression/ Delinquency T2	1.83 (2.41)	1.94 (1.77)	0.37 (0.29–0.43)***
Aggression/ Delinquency T3	0.94 (1.71)	1.82 (1.77)	0.10 (0.01–0.19)**

*M* mean, *SD* standard deviation, *ICC* intraclass correlation.

*P* values > F: *p < 0.05; **p < 0.01; ***p < 0.001. *N* = 675.

**Table 4:** Descriptive statistics and intercorrelations between variables included into the Path Model 1 (teacher rating).

		1	2	3	4	5	6	7	8	M	SD	Min	Max
1	Prematurity/SGA	1								–	–	–	–
2	Teacher: Hyperactivity/ Inattentiveness T1	0.09^*^	1							3.76	3.96	0	16
3	Teacher: Hyperactivity/ Inattentiveness T2	0.08*	0.75^***^	1						3.39	3.94	0	16
4	Teacher: Hyperactivity/ Inattentiveness T3	0.05	0.46^***^	0.40^***^	1					3.16	3.87	0	16
5	Teacher: Aggression/ Delinquency T1	0.03	0.58^***^	0.52^***^	0.39^***^	1				1.83	2.27	0	12
6	Teacher: Aggression/ Delinquency T2	0.02	0.48^***^	0.62^***^	0.38^***^	0.74^***^	1			1.83	2.41	0	12
7	Teacher: Aggression/ Delinquency T3	0.03	0.37^***^	0.32^***^	0.58^***^	0.44^***^	0.43^***^	1		0.94	1.71	0	11
8	Sex	–0.05	–0.26^***^	–0.31^***^	–0.35^***^	–0.36^***^	–0.45^***^	–0.32^***^	1	–	–	–	–

*M* mean, *SD* standard deviation, *Min* minimum, *Max* maximum.

*p < .05; **p < .01; ***p < .001. *N* = 667.

**Table 5:** Descriptive statistics and intercorrelations between variables included into the Path Model 2 (parent rating).

		1	2	3	4	5	6	7	8	M	SD	Min	Max
1	Prematurity/SGA	1								–	–	–	–
2	Parent: Hyperactivity/ Inattentiveness T1	0.11^**^	1							4.93	3.47	0	16
3	Parent: Hyperactivity/ Inattentiveness T2	0.14***	0.72^***^	1						4.64	3.67	0	16
4	Parent: Hyperactivity/ Inattentiveness T3	0.15***	0.65^***^	0.74^***^	1					4.42	3.44	0	16
5	Parent: Aggression/ Delinquency T1	0.04	0.44^***^	0.33^***^	0.30^***^	1				2.27	1.87	0	11
6	Parent: Aggression/ Delinquency T2	0.08*	0.434^***^	0.41^***^	0.34^***^	0.62^***^	1			1.94	1.77	0	9
7	Parent: Aggression/ Delinquency T3	0.07	0.29^***^	0.31^***^	0.41^***^	0.58^***^	0.67^***^	1		0.82	1.77	0	9
8	Sex	–0.05	–0.14^***^	–0.16^***^	–0.16^***^	–0.25^***^	–0.30^***^	–0.26^***^	1	–	–	–	–

*M* mean, *SD* standard deviation, *Min* minimum, *Max* maximum.

*p < 0.05; **p < 0.01; ***p < 0.001. *N* = 667.

**Fig. 1:**
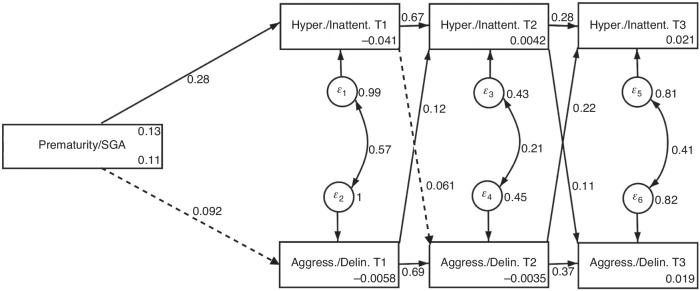
Path Model 1 with direct effects (teacher rating). SGA small for gestational age. Prematurity/SGA: 0 = not present/1 = present. Significant paths displayed as solid lines; non-significant paths displayed as dashed lines. *N* = 667.

Figure 1 displays the predicted Path Model 1 based on teacher ratings with the direct effects of the path coefficients. The results show direct effects of birth complications at the beginning of preschool on hyperactivity (*ß* = 0.28; *p* = 0.017), but not on aggression/delinquency (*ß* = 0.092; *p* = 0.427). There were significant effects of aggression/delinquency on hyperactivity/inattentiveness at the end of elementary school (*ß* = 0.11; *p* = 0.039). Effects of hyperactivity/inattentiveness on aggression/delinquency at the end of preschool, however, only reached significance at a 10% level (*ß* = 0.061; *p* = 0.064). Effects of aggression/delinquency on hyperactivity/inattentiveness were stable at both timepoints, the end of preschool (*ß* = 0.12; *p* = 0.000), and at the end of elementary school (*ß* = 0.22; *p* = 0.000) and increased over time.

Autoregressive effects for both, hyperactivity/ inattentiveness as well as aggression/delinquency were significant and decreased over time for both, hyperactivity/inattentiveness (*ß* = 0.67 *p* = 0.000; *ß* = 0.28; *p* = 0.000) as well as for aggression/delinquency (*ß* = 0.69 *p* = 0.000; *ß* = 0.37; *p* = 0.000). A good model fit to the data was provided (see Table 6).

**Table 6:** Goodness of fit indices for Path Model 1 (teacher rating).

Fit statistics	*Chi*^*2*^ *(df)*	*p*	RMSEA	CFI	TLI
Values	51.87 (8)	0.000	0.091	0.976	0.937

*Chi*^*2*^ Chi-square statistic, *df* degrees of freedom, *RMSEA* Root Mean Square Error of Approximation, *CFI* Comparative Fit Index, *TLI* Tucker-Lewis Index.

In a next step, we tested our Model 1 based on teacher ratings for subsamples consisting of only boys and only girls respectively. Path coefficients for Model 1.1 that includes only boys are displayed in Table 7. Consistent with the overall Model 1, significant direct effects are found for boys of birth complications on hyperactivity/inattentiveness at the beginning of preschool (*ß* = 0.347; *p* = 0.029), but not on aggression/delinquency at preschool (*ß* = 0.081 *p* = 0.619). Hyperactive/inattentive behavior at the end of preschool is predicted by hyperactive/inattentive behavior at the beginning of preschool (*ß* = 0.644; *p* = 0.000) and aggressive/delinquent behavior at the beginning of preschool (*ß* = 0.129; *p* = 0.009). Hyperactive/inattentive behavior at the end of elementary school is predicted by hyperactive/inattentive behavior at the end of preschool (*ß* = 0.314; *p* = 0.000) and aggressive/delinquent behavior at the end of preschool (*ß* = 0.146; *p* = 0.035).

**Table 7:** Effects in the Path Model 1.1 (teacher rating) including only boys and the Path Model 1.2 (teacher rating) including only girls.

	Path Model 1.1 - Boys		Path Model 1.2 - Girls	
Variable	*ß*	*p*	*ß*	*p*
Hyperactivity/Inattentiveness T1 ←				
Prematurity/SGA	0.347	0.029	–0.047	0.765
Aggressive behavior/Delinquency T1 ←				
Prematurity/SGA	0.081	0.619	–0.100	0.484
Hyperactivity/Inattentiveness T2 ←				
Hyperactivity/Inattentiveness T1	0.644	0.000	0.678	0.000
Aggressive behavior/Delinquency T1	0.129	0.009	0.031	0.463
Aggressive behavior/Delinquency T2 ←				
Hyperactivity/Inattentiveness T1	0.019	0.703	0.073	0.030
Aggressive behavior/Delinquency T1	0.714	0.000	0.490	0.000
Hyperactivity/Inattentiveness T3 ←				
Hyperactivity/Inattentiveness T2	0.314	0.000	0.245	0.001
Aggressive behavior/Delinquency T2	0.147	0.035	0.010	0.912
Aggressive behavior/Delinquency T3 ←				
Hyperactivity/Inattentiveness T2	0.139	0.107	0.072	0.136
Aggressive behavior/Delinquency T2	0.311	0.000	0.222	0.001

Path Model 1.1 (boys): *Chi*^*2*^ *(df)* = 38.31 (8); *p* = 0.000; *RMSEA* = 0.107; *CFI* = 0.962; *TLI* = 0.900; *N* = 234. Path Model 1.2 (girls): *Chi*^*2*^ *(df)* = 10.00 (8); *p* = 0.265; *RMSEA* = 0.027; *CFI* = 0.996; *TLI* = 0.993; *N* = 333.

However, aggressive/delinquent behavior is not predicted by hyperactive/inattentive behavior, neither at the end of preschool (*ß* = 0.019; *p* = 0.703), nor at the end of elementary school (*ß* = 0.139; *p* = 0.107). Autoregressive effects for hyperactive/inattentive behavior (*ß* = 0.644; *p* = 0.000; *ß* = 0.314; *p* = 0.000) as well as for aggressive/delinquent behavior (*ß* = 0.714; *p* = 0.000; *ß* = 0.311; *p* = 0.001) are stable for boys.

Path coefficients for Model 1.2 that includes only girls are also displayed in Table 7. As opposed to the overall Model 1 as well as Model 1.1, no direct effects are found for girls of birth complications on hyperactivity/inattentiveness at the beginning of preschool (*ß* = –0.047; *p* = 0.765), nor on aggression/delinquency at preschool (*ß* = –0.100; *p* = 0.484). There are no stable significant reciprocal effects between hyperactivity/inattentiveness and aggression/delinquency for girls. Only aggressive/delinquent behavior at the end of preschool was significantly predicted by hyperactive/inattentive behavior at the beginning of preschool (*ß* = 0.073; *p* = 0.030). Autoregressive effects for hyperactive/inattentive behavior (*ß* = 0.678; *p* = 0.000; *ß* = 0.245; *p* = 0.001) as well as for aggressive/delinquent behavior (*ß* = 0.490; *p* = 0.000; *ß* = 0.222; *p* = 0.001) are stable for girls.

**Fig. 2:**
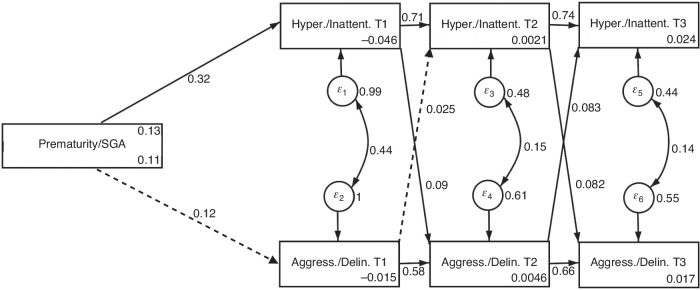
Path Model 2 with direct effects (parent rating). SGA small for gestational age. Prematurity/SGA: 0 = not present/1 = present. Significant paths displayed as solid lines; non-significant paths displayed as dashed lines. *N* = 667.

Figure 2 displays a replication of the path model based on parent ratings (Model 2) with the direct effects of the path coefficients. In accordance with Model 1, direct effects exist of birth complications on hyperactivity at the beginning of preschool (*ß* = 0.324; *p* = 0.005), but not on aggression/delinquency (*ß* = 0.12; *p* = 0.324). As opposed to Model 1, there were significant effects of hyperactivity/inattentiveness on aggression/delinquency at the end of preschool school (*ß* = 0.09; *p* = 0.009). In turn, effects of aggression/delinquency on hyperactivity/inattentiveness at the end of preschool did not reach significance (*ß* = 0.025; *p* = 0.409).

Autoregressive effects for both, hyperactivity/ inattentiveness as well as aggression/delinquency were significant and increased over time for both, hyperactivity/inattentiveness (*ß* = 0.71 *p* = 0.000; *ß* = 0.74; *p* = 0.000) as well as for aggression/delinquency (*ß* = 0.58; *p* = 0.000; *ß* = 0.66; *p* = 0.000). A good model fit to the data was provided (see Table 8).

**Table 8:** Goodness of fit indices for Path Model 2 (parent rating).

Fit statistics	Chi^2^ (df)	*p*	RMSEA	CFI	TLI
Values	97.17 (8)	0.000	0.129	0.954	0.879

*Chi*^*2*^ Chi-square statistic, *df* degrees of freedom, *RMSEA* Root Mean Square Error of Approximation, *CFI* Comparative Fit Index, *TLI* Tucker-Lewis Index.

Path coefficients for Model 2.1 including only boys and Model 2.2 including only girls are displayed in Table 9. Results show a stable significant effect of birth complications on hyperactivity/inattentiveness at the beginning of preschool for boys (*ß* = 0.36; *p* = 0.020), but not for girls (*ß* = 0.23 *p* = 0.193).

**Table 9:** Effects in the Path Model 2.1 (parent rating) including only boys and the Path Model 2.2 (parent rating) including only girls.

	Path Model 2.1 - Boys		Path Model 2.2 - Girls	
Variable	*ß*	*p*	*ß*	*p*
Hyperactivity/Inattentiveness T1 ←				
Prematurity/SGA	0.361	0.020	0.228	0.193
Aggressive behavior/Delinquency T1 ←				
Prematurity/SGA	0.208	0.182	–0.087	0.618
Hyperactivity/Inattentiveness T2 ←				
Hyperactivity/Inattentiveness T1	0.713	0.000	0.713	0.000
Aggressive behavior/Delinquency T1	0.035	0.392	–0.018	0.685
Aggressive behavior/Delinquency T2 ←				
Hyperactivity/Inattentiveness T1	0.106	0.032	0.068	0.157
Aggressive behavior/Delinquency T1	0.531	0.000	0.607	0.000
Hyperactivity/Inattentiveness T3 ←				
Hyperactivity/Inattentiveness T2	0.725	0.000	0.728	0.000
Aggressive behavior/Delinquency T2	0.048	0.266	0.107	0.010
Aggressive behavior/Delinquency T3 ←				
Hyperactivity/Inattentiveness T2	0.007	0.869	0.174	0.000
Aggressive behavior/Delinquency T2	0.672	0.000	0.572	0.838

Path Model 2.1 (boys): *Chi*^*2*^ *(df)* = 53.98 (8); *p* = 0.000; *RMSEA* = 0.131; *CFI* = 0.948 *TLI* = 0.864; *N* = 334. Path Model 2.2 (girls): *Chi*^*2*^ *(df)* = 53.98 (8); *p* = 0.000; *RMSEA* = 0.131; *CFI* = 0.948 *TLI* = 0.864; *N* = 334.

## Discussion

This study adds to the understanding of the complex interplay between prematurity and growth restrictions as birth complications and hyperactive/inattentive and aggressive/delinquent behavior problems in early, middle, and late childhood. In this study, birth complications were considered as antecedent factors that may influence the emergence of hyperactive/inattentive and aggressive/delinquent problems in later childhood. Associations were examined in path models based on teacher respectively parent ratings. We analyzed cross-lagged effects between both forms of behavioral problems, taking into account the stability of hyperactive/inattentive and aggressive/delinquent behavior problems over time.

For our total sample, we found direct effects of birth complications on hyperactivity/inattentiveness, but not on aggression/delinquency at the beginning of preschool. This result was replicated based on teacher as well as on parent ratings. This finding is in line with previous results, indicating that birth complications have an impact on neurological development, leading to deficits in executive functions, impulsivity or less self-control.^8,11,15–18^ Direct effects of birth complications, such as premature birth and growth restrictions on hyperactive behavior are reported by a number of studies.^29–31,33,36^ Our results are in line with this body of research.

For our total sample, we found stable reciprocal associations between aggression/delinquency and hyperactivity/inattentiveness at the end of elementary school for both, teacher and parent ratings. This finding is in line with psychological theory, as aggression and other disruptive behaviors often co-occur with inattention and hyperactivity.^58–60^ Autoregressive effects for hyperactive/inattentive behavior as well as for aggressive/delinquent behavior are also stable based on both, teacher and parent ratings. Similar findings are reported by other studies that examined autoregressive effects of hyperactive.^61^ and externalizing behavior.^62^

However, we did not find fully consistent associations between hyperactivity/inattentiveness and aggression/delinquency across all time points of measurement. Based on the teacher rating, the effects of hyperactivity/inattentiveness on aggression/delinquency at the end of preschool only reached significance at a 10% level, whereas aggression/delinquency significantly predicted hyperactivity/inattentiveness. Based on the parent rating, associations between hyperactivity/inattentiveness and aggression/delinquency at the end of preschool presented in reverse direction.

Some theoretical considerations argue that birth complications may not have a direct effect on the emergence of aggressive behavior in later childhood but might be effective via hyperactivity. Childhood hyperactivity may in turn be linked to juvenile aggression and criminal behavior.^19–22^ This assumption is only supported by our results based on the parent, but not teacher rating, especially for younger children at preschool age.

When replicating our models for the subsample consisting of boys only, consistent with the overall model, we found significant direct effects of birth complications on hyperactivity/inattentiveness at the beginning of preschool, but not on aggression/delinquency at preschool for both teacher and parent ratings. When replicating our model for the subsample consisting of girls only, we found neither direct effects of birth complications on hyperactivity/inattentiveness at the beginning of preschool, nor on aggression/delinquency at preschool for both teacher and parent ratings. Reciprocal effects between hyperactivity/inattentiveness and aggression/delinquency were non-significant for girls. Autoregressive effects of hyperactive/inattentive behavior as well as aggressive/delinquent behavior over time were stable for both boys and girls across informants.

Effects of the sex of the child on the association between birth complications and behavioral problems are reported by a number of studies. Jackson and Vaughn.^32^ found that associations between a greater number of maternal medical risks during pregnancy and childhood externalizing behavior were only found for boys, but not for girls.^32^ Mollegaard.^33^ report that birth weight was significantly negatively associated with hyperactivity at age 12, but only for boys, not for girls.^33^ Taylor et al.^36^ also report effects of the sex of the child on the association between extremely low birth weight and ADHD, as group differences in parent ratings decreased over time for ADHD, especially among females.^36^

Therefore, our findings add to this body of research, supporting the link between prematurity and growth restrictions as birth complications and the emergence of hyperactivity/inattentiveness across early, middle, and late childhood for male, but not for female children. This finding is in line with the literature that shows that girls exhibit much less externalizing but more internalizing problems, like anxious or depressive symptoms.^34,63^ However, even for boys, our results provide mixed support for the hypothesis that birth complications have an indirect effect on the emergence of aggressive and delinquent behavior in boys via hyperactivity/inattentiveness at preschool and elementary school age.

Why our study revealed a predictive effect of the theoretically mediating construct of hyperactivity/inattentiveness on aggression/delinquency only based on parent ratings may to some extend be due to methodological limitations. This finding may partially be explained by the fact that our sample mostly consists of children from families in a relatively prosperous region of Germany with rather low unemployment and good access to medical care. Rates for prematurity in our sample are below general preterm birth rates estimates for Germany, which were between 8 to 9% for the last decades.^47^ Estimated low birthweight rates for Germany were between 6.5 to 6.6% over the last decades,^64^ and rates found in our sample were also lower. Results on health-related variables at birth overall indicate that children in our sample were born rather healthy. This may have led to only few extreme cases in our sample, both in terms of birth complications and behavior problems, which may have contributed to variance limitations (i.e., when reducing our sample size by including boys/girls only).

Another explanation might lie within using preschool and elementary school teacher’s ratings to analyze longitudinal relations. Although this is a strength of our study (see below), due to their professional experience, inattention and hyperactivity may have been perceived as less serious than aggressive and delinquent behavior of the children.

Furthermore, it should be taken into account that at our last time point of measurement, children were at the end of elementary school age. As delinquency typically increases at a later age in adolescence or young adulthood, this is both a strength and limitation to our analysis. It is a strength because there may have been relatively homogenous biological and social conditions before puberty. Afterwards, biological changes and increasing detachment from the family as well as more influence of peers may lead to more complicated patterns of influences.^34^ Research suggests that peer influences become most relevant for deviant behavioral development in youth.^65–67^ what may reduce the influence of early neuropsychological dispositions. We are planning to address this issue in the future.

### Limitations, strengths, and practical implications

The results of the present study should be considered alongside several limitations. We only considered birth complications as antecedent factors that have an influence on the emergence of hyperactive/inattentive and aggressive/delinquent behavior problems. However, birth complications might interact with family risks, such as inadequate parenting behavior, to predict behavioral problems.^8,13,15,37,38^ Family factors like parenting and social support can have both an additional risk or a protective effect.

Given that in our study design, information on birth complications were obtained via medical records when the children were between birth and preschool age, we did not have access to information on further biosocial factors that were not included in the booklets of pediatric assessments. This refers, for example, to cases of mothers’ substance use in pregnancy that may have led to a fetal alcohol syndrome, or to children’s health status throughout their later development, including for example chronic diseases or accidents. The focus of our study could only highlight the specific impact of birth complications on hyperactive/inattentive and aggressive/delinquent problems during childhood. Following a scientifically parsimonious approach, more influences and comorbidity problems were not assessed to avoid a reduction of statistical power due to many variables. Broadening our understanding on which additional factors enhance or buffer these associations is open to future research.

It is a strength of our study that, in addition to the parents, preschool and schoolteachers assessed the behavior of the children, because in most cases they would not have had knowledge about birth complications of the children. Only in a few cases – for example when children were born very preterm - the parents may have told teachers about it, but this may rarely have been the case. In this respect, the collection of data from routine pediatric exams and the later behavioral assessments by preschool and elementary school teachers provide highly independent data. As we included different teacher’s ratings, this may have reduced the influence of stereotypes and stigmatization.

It is also a strength that the teachers’ assessments correlated significantly with the child ratings by the parents. These relations were in the typical range found in the literature for child assessments in different contexts. Intrarater-agreement between parents and teachers on behavioral variables in our sample was low to moderate, what is in line with the literature.^52,53,68,69^ Various studies have shown that the correlations between different informants on child behavior problems in different contexts are typically low to moderate.^50,68^

Furthermore, it must be taken into account that the data used on birth complications originate from routine pediatric exams. Although these can be considered relatively objective, they do not provide detailed information about serious potential neuropsychological impairments. This would have to be assessed more directly in clinical studies.

We were able to identify far-reaching effects of birth complications on the emergence of hyperactive and inattentive behavior from early through late childhood, especially for boys. However, it remains open how long these effects will last. As mentioned, the transition from childhood to adolescence is a critical period. With the exception of extreme cases, behavior development is rather flexible and the majority of children with early externalizing problems may develop no long-term deviance.^70^

In spite of its limitations, our study is relevant for practice. Our results support realistic and anxiety reducing information of parents with regard to potential consequences of birth complications for child behavioral development. They also support the demand for early prevention measures for externalizing behavioral problems, especially hyperactivity and inattentiveness.^71,72^ Tremblay.^73^ recommends starting preventive measures in early pregnancy. The successful Nurse-Family Partnership Program.^74,75^ also points in this direction. Health-promoting interventions of pregnant women to reduce pregnancy and birth complications could reduce the likelihood of externalizing behavior problems already at a very early stage.^11^ In Germany, so called “early help” programs (Fruehe Hilfen) have been anchored in the German Federal Child Protection Act since 2012, offering a basis for such interventions. International reviews clearly showed significantly positive results of family-oriented prevention programs.^72^ as well as child-oriented social competence trainings.^76^

## Data Availability

The datasets analyzed in the current study are available from the corresponding author on reasonable request.
